# Biomechanical Determinants of Knee Joint Loads Associated with Increased Anterior Cruciate Ligament Loading During Cutting: A Systematic Review and Technical Framework

**DOI:** 10.1186/s40798-020-00276-5

**Published:** 2020-11-02

**Authors:** Thomas A. Donelon, Thomas Dos’Santos, Guy Pitchers, Mathew Brown, Paul A. Jones

**Affiliations:** 1 Room Af87, Section of Sport and Exercise Sciences, School of Human and Life Sciences, Canterbury Christ Church University, North Holmes Road, Canterbury, Kent CT1 1QU UK; 2 Department of Sport and Exercise Science, Manchester Metropolitan University, Bonsall Street, Manchester, M15 6GX UK; 3 School of Health Sciences, University of Salford, C702 Allerton Building, Salford, M6 6PU UK

**Keywords:** ACL, Knee joint loads, Sidestepping, Technical Framework, Injury-Performance Conflict

## Abstract

**Background:**

Cutting actions are associated with non-contact ACL injuries in multidirectional sports due to the propensity to generate large multiplanar knee joint loads (KJLs) that have the capacity to increase ACL loading and strain. Numerous studies have investigated the biomechanical determinants of KJLs in cutting tasks. The aim of this systematic review was to comprehensively review the literature regarding biomechanical determinants of KJLs during cutting, in order to develop a cutting technical framework alongside training recommendations for practitioners regarding KJL mitigation.

**Methods:**

Databases (SPORTDiscus, Web of Science and PubMed) were systematically searched using a combination of the following terms: “Biomechanical determinants”, or “Knee abduction moment”, or “Technical determinants”, or “Knee loading”, or “Knee loads”, or “Mechanical determinants”, or “ACL strain”, or “Knee adduction moment”, or “Anterior tibial shear”, or “Knee internal rotation moment”, or “Knee valgus moment” AND “Change of direction”, or “Cutting manoeuvre”, or “Run and cut”, or “Run-and-cut”, or “Sidestepping”, or “Side-stepping”, or “Shuttle run”. Inclusion criteria were as follows: studies examining a cutting task < 110° with a preceding approach run that examined biomechanical determinants of KJLs using three-dimensional motion analysis.

**Results:**

The search returned 6404 possibly eligible articles, and 6 identified through other sources. Following duplicate removal, 4421 titles and abstracts were screened, leaving 246 full texts to be screened for inclusion. Twenty-three full texts were deemed eligible for inclusion and identified numerous determinants of KJLs; 11 trunk, 11 hip, 7 knee, 3 multiplanar KJLs, 5 foot/ankle and 7 identifying ground reaction forces (GRFs) as determinants of KJLs.

**Conclusion:**

Using the framework developed from the results, cutting KJLs can be mitigated through the following: reducing lateral foot-plant distances, thus lowering hip abduction and orientating the foot closer to neutral with a mid-foot or forefoot placement strategy; minimising knee valgus and hip internal rotation angles and motion at initial contact (IC) and weight acceptance (WA); avoiding and limiting lateral trunk flexion and attempt to maintain an upright trunk position or trunk lean into the intended direction; and finally, reducing GRF magnitude during WA, potentially by attenuation through increased knee flexion and emphasising a greater proportion of braking during the penultimate foot contact (PFC).

## Key Points

- High-risk postures in cutting include frontal plane trunk alignment away from the cutting direction, an internally rotated hip; initial valgus alignment of the knee at weight acceptance; a knee close to full extension; wide foot-plant distance (analogous with hip abduction); increased foot progression angle (inverted foot towards the midline), rearfoot landings and a lack of PFC braking strategy.
- Practitioners are encouraged to coach a reduction in lateral foot-plant distances, reducing hip abduction and orientating the foot closer to neutral with a mid-foot strike strategy. Coupling this with a minimisation of knee valgus and hip internal rotation at initial contact (IC) and weight acceptance (WA) alongside encouraging trunk lean in the intended direction should ameliorate KJLs. An attempt to reduce the magnitude of the GRF in the final foot contact should be made, through emphasising a greater proportion of braking in the prior steps to turning.
- Practitioners should consider training strategies which reduce high-risk postures and biomechanics, both of which are modifiable risk factors for potentially hazardous multiplanar knee loads.

## Background

Non-contact anterior cruciate ligament (ACL) injuries are one of the most devastating injuries an athlete can sustain. They occur in a multitude of sports [1–3], typically during high-impact tasks such as decelerations, landing and changes of direction (CODs), namely cutting [1, 2, 4–8]. There are a large number of ACL injuries reported annually (80,000 per year in the USA alone) [9], 70% arising from non-contact situations [10] with an incidence rate of 0.42 per 1000 player hours [11]. Such injuries carry with them a return to play period of approximately 6–24 months [12, 13] and are associated with an increased risk in the development of osteoarthritis in later life [14]. Furthermore, the estimated cost of ACL reconstructions in America is estimated to be 17,000 dollars per reconstruction [9], highlighting the financial burden they can place on healthcare and public health services, thus providing rationale for their mitigation.

ACL injuries occur when a load exceeds the ligament’s tolerance [15]. Cadaver [16–18] and computer simulated modelling studies [19, 20] have examined ACL strain and forces to gain a greater understanding of the respective biomechanical parameters responsible. Externally applied knee abduction moments (KAMs) and knee internal rotation moments (KIRMs), alongside anterior tibial shear force (ASF), have all been identified as loading parameters that have the potential to increase ACL strain [17, 18, 20, 21] and are subsequently used as surrogates of non-contact ACL injury risk. McLean et al. [22] suggest that uniplanar loading in the sagittal plane is insufficient to elicit ACL rupture, elsewhere the mechanism of ACL injury has been described as multiplanar [23, 24]. Unsurprisingly, a plethora of studies have identified multiplanar knee joint loads (KJLs) to elicit significantly greater ACL strain than uniplanar loads alone [17, 18, 21]. Recently, Bates et al. [18] identified KAMs as eliciting the greatest change in ACL strain in isolated conditions than ASF or tibial internal rotation. However, the greatest level of ACL strain observed was when all three loading parameters were applied in the greatest magnitude conditions, substantiating previous evidence of multiplanar loading being the most potent stimulus for ACL strain [16, 17, 21].

COD tasks such as cutting manoeuvres (which for the purpose of this review will be referred to as changes of direction between 30° and 110°) are key actions associated with non-contact ACL injuries; thus, understanding the biomechanical determinants of multiplanar KJLs in cutting is of great importance, particularly for athletes who participate in cutting dominant sports. Cutting has retrospectively been identified as the primary action associated with non-contact ACL injury in rugby (66% [7];) American football (60% [5];) and handball (60% [2];). Furthermore, cutting has been found to elicit greater multiplanar KJLs than both single legged landings (SLLs) [25] and multiplanar side jumps amongst athletes [26]. When considering the large number of cuts in invasion games [27, 28], this creates a problem for health practitioners and athletic development staff, whereby athletes are being continuously exposed to potentially injurious loads. Therefore, understanding the technical and mechanical determinants of these loads in cutting tasks is of paramount importance to inform appropriate injury mitigation practice.

In order to better understand the mechanism of non-contact ACL injuries, several studies have identified several key postures during visual observations of non-contact ACL injuries. For example, contralateral trunk lean has been observed in numerous ACL incidents in female athletes [29]. Frontal and sagittal hip motion whereby an abducted and flexed hip has been observed in 86% and 96% (respectively) of non-contact ACL injuries in a cohort of National Football League (NFL) players across three seasons [5]. Additionally, a knee posture that is close to full extension is a prominent characteristic in non-contact ACL ruptures in American football [5], elite rugby [7] and handball [7]. Knee abduction was observed in 11/12 non-contact ACL incidents in handball (with tibial rotation) [2], 92% of non-contact ACL injuries in American Football [5] and in injured basketball players (28.7° ± 19.5° greater in injury incidents) [4]. Regarding the foot, a rearfoot landing was a consistently observed feature in rugby (90% of cases: *p* < 0.001) [7] and basketball [4]. Finally, an everted ankle/foot position has been observed in 90% of non-contact ACL injuries in the NFL [5]. All of the above detail a phenomenon first described by Hewett as “dynamic valgus” [30] that has been identified as a contributory movement pattern in non-contact ACL injuries.

The apparent consistencies between non-contact ACL rupture incidents suggest that these commonly observed technical postures are contributory to the biomechanical load elicited at the knee, leading to rupture. A plethora of research has investigated these “biomechanical determinants” (i.e. body postures and kinetics that determine the magnitude of KJLs) in order to advise practitioners what is best regarding optimal technique to reduce multiplanar KJLs in cutting [31–37]. However, these studies have used varying cutting angles, approach velocities and cohort of athlete all which can affect multiplanar KJLs [32, 38, 39] making between-study comparisons difficult. Numerous intervention studies have attempted to reduce KJLs through targeting these segments that contribute to multiplanar KJLs with mixed success [31, 40, 41]. Dempsey et al. elicited 36% reduction in KAMs through modifying contralateral trunk lean and foot-plant width. Adopting this multi-segmental approach may be more beneficial, considering other uni-segmental interventions have failed to elicit any reduction in KJLs [40, 41]. Furthermore, these intervention studies [31, 40, 41] have failed to measure any possible detrimental effect upon performance which would have a succinct effect on engagement and adherence to an intervention, as athletes will not engage with an intervention that will elicit performance decrements [34].

Previous reviews have commentated on risk factors associated with cutting and non-contact ACL injury risk [42, 43], although these have been narrative and not developed guidelines for practitioners. To date, no technical model for cutting exists. Therefore, to inform effective and adhered to injury mitigation protocols, the construction of a research informed technical model detailing optimal cutting technique would be beneficial for practitioners working with athletes in cutting dominant sports. This would provide a better understanding of the “high-risk” postures and mechanics that increase KJLs during cutting and subsequent ACL loading and provide clarity on safe cutting technique. Therefore, the aim of this systematic review is twofold: firstly, to comprehensively review the literature available regarding biomechanical determinants of KJLs in cutting tasks and factors affecting them with regard to injury and performance; and secondly, to develop a technical framework alongside recommendations for practitioners regarding mitigations of KJLs during cutting tasks.

## Methods

### Searches

A literature search was performed using PubMed, Web of Science and SPORTDiscus databases from January 2019 to May 2020 with the final search date of 5 May 2020. A schematic of search methodology in accordance with established guidelines [44] will be presented in the results below. Search terms were as follows:

(1) “Biomechanical determinants”, or “Knee abduction moment”, or “Technical determinants”, or “Knee loading”, or “Knee loads”, or “Mechanical determinants”, or “ACL strain”, or “Knee adduction moment”, or “Anterior tibial shear”, or “Knee internal rotation moment”, or “Knee valgus moment” AND

(2) “Change of direction”, or “Cutting manoeuvre”, or “Run and cut”, or “Run-and-cut”, or “Sidestepping”, or “Side-stepping”, or “Shuttle run”

Subsequently bibliographies of prospectively eligible studies were compiled and hand searched to screen for further suitable studies. If disagreement occurred between the two reviewers (TD and TDS), a third independent reviewer (PJ) was consulted and their decision deemed as final.

### Study Inclusion and Exclusion Criteria

Inclusion criteria for studies were as follows:

1. Investigated a pre-planned or unplanned cutting task that contained a preceding approach run and subsequent change in direction. The decision was made to omit tasks that included a false step or hop or that omitted an approach run. Omission of an approach run would not truly replicate the loading parameters of non-contact ACL injury situations due to the absence of a deceleration (which has been identified as the component where most non-contact ACL ruptures occur) and redirection component that are asymptomatic of horizontal velocity. Cutting tasks of up to 110° were considered for inclusion as it was deemed that any COD greater than this (i.e. pivoting actions) would possess succinct biomechanical differences that would require a different technical framework.
2. Examined technical or biomechanical determinants of knee joint moments or loads in a cutting task using 3D motion and GRF analysis including the effect of exercise intervention or technique modification.
3. Included healthy participants with no previous history of ACL injury.
4. Original research, full text published in a peer review journal, in English.

Studies that did not meet the abovementioned criteria were excluded from the review.

### Assessment of Study Quality

An assessment of study quality was conducted as per previously established methods [45, 46] using the modified scale constructed by Brown et al. [46]. This is deemed to be more suitable for assessing the methodological quality of COD studies due to the omission of criteria such as random allocation, assessor blinding and subject blinding that are present in more commonly used scales such as the Cochrane or Delphi, Physiotherapy, Evidence Database scales. Each component was individually scored from 0 to 2 (where 0 = clearly no, 1 = maybe or insufficient information and 2 = clearly yes).

### Data Collation

Data were independently extracted from each study by the lead reviewer (TD). Results were collated through identifying significant findings (*p* < 0.05), correlational *R* values and coefficients of determination (*R*^2^). Data were then systematically separated by cutting angle investigated and the subsequent biomechanical determinant/segment that was cited by each respective study. Data was sought for all primary outcome measures which were multiplanar KJLs. Peak variables obtained from discrete point analysis methods, and variables derived from continuous analysis methods such as statistical parametric mapping (SPM) were sought for all primary outcome measures. The external and internal (respectively) joint moment conventions were all searched for and included the following: knee abduction moment (KAM), knee adduction moment (KADM), knee flexion moment (KFM), knee extension moment (KEM), and knee internal rotation moment (KIRM).

## Results

### Search Results

**Fig. 1:**
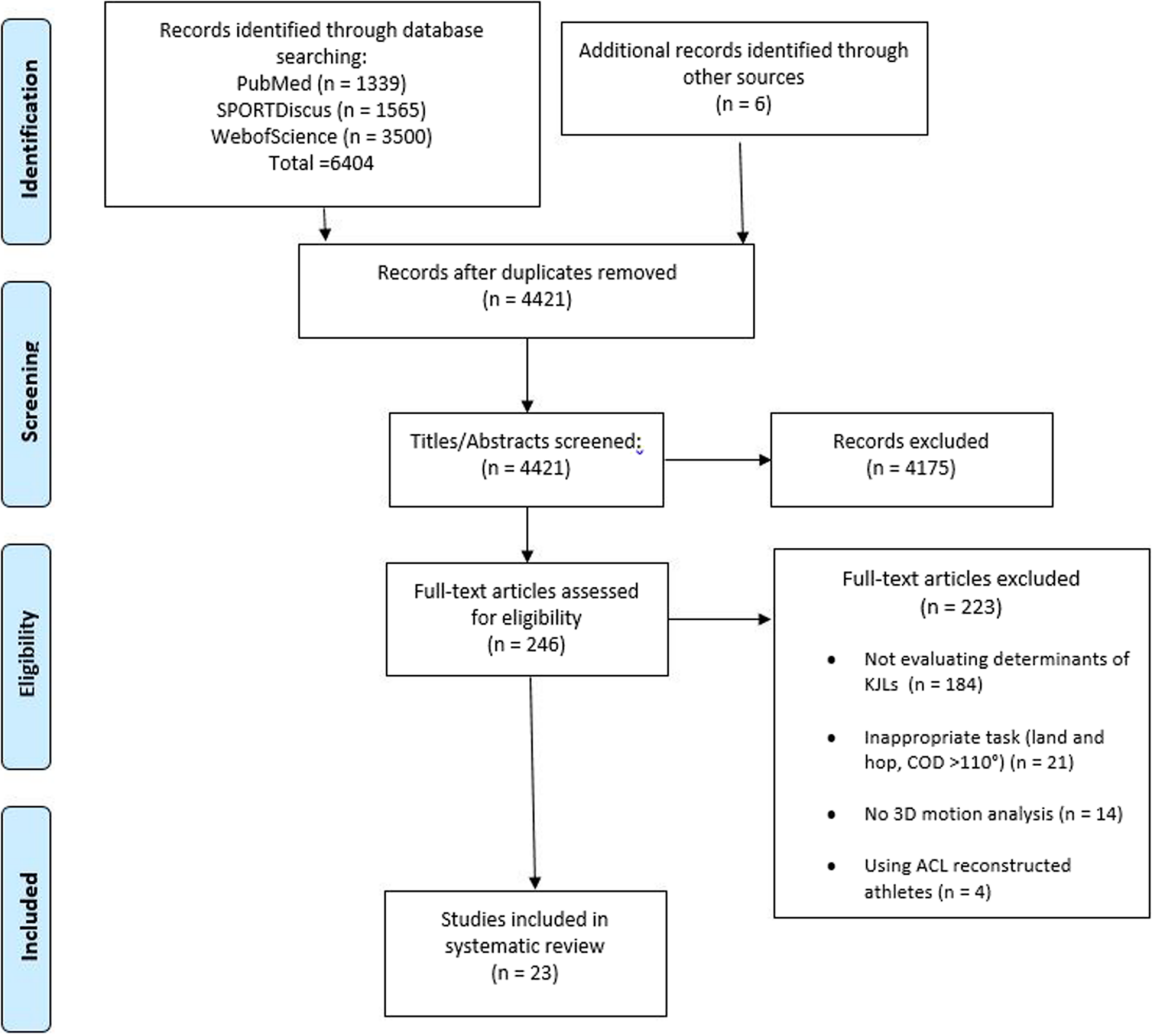
Flow chart illustrating the different phases of the search strategy and study selection process. Key: 3D: three-dimensional; GRF: ground reaction force; KJL: knee joint load; ACL: anterior cruciate ligament

Figure 1 presents a flow chart summarising the results of the systematic search process whereby 6404 potentially eligible articles were identified across PubMed, Web of Science and SPORTDiscus databases. Following title, abstract and full text screening, 17 studies were deemed eligible for inclusion in this review. A further 6 eligible studies were identified from the reference list screening of eligible studies. Of the 23 studies deemed eligible for inclusion, 15 [31, 34, 35, 37, 38, 40, 41, 47–54] examined a 45° cut; six [34, 36, 55–58] a 90° cut; three involved self-selected ranges of 35°–60° [59] and 70°–90° [60] and a mean cutting angle of 67° [61], and one utilised a 110° cut [37]. With regard to anticipation, 12 studies [34, 36, 38, 48, 51, 53, 55–58, 60, 61] adopted a pre-planned cut, with eight [35, 37, 40, 41, 47, 49, 50, 54] utilising unplanned cuts; three studies [31, 52, 59] examined both pre-planned and unplanned cuts. Regarding the approach velocity of tasks, five studies adopted a maximum effort approach [34, 56, 57, 60, 61], with four utilising a self-selected pace [35, 40, 50, 55]. Seven studies utilised approach velocity windows [36, 37, 49, 51, 53, 54, 58] ranging from 3.5–4.5 m/s [54] to 5.5–7.0 m/s [53]. Six studies [31, 41, 47, 48, 59, 62] utilised approach velocities ranging from 3.5 [55] to 5.2 m/s [31]. Finally, one study adopted varying incremental approach speeds of 2, 3, 4 and 5 m/s [38]. All 23 studies examined KAMs, with three examining KIRMS [35, 54, 60] and two examining KFMs (external)/KEMs respectively [54, 60].

### Assessment of Methodological Quality

In accordance with the methods of Dos’Santos et al. [45] and Brown et al. [46], assessment of methodological quality was conducted on the 23 studies deemed eligible for inclusion in this review and is presented in Table 1. Of the 23 studies, the mean methodological quality score was 11.86 (66%). Scores ranged from 6 (33%) [35] to 16 (88%) [60]. Eleven studies were below this mean score [35, 36, 38, 49–51, 54–56, 58, 61], with 12 studies presenting methodological quality greater than the mean [31, 34, 37, 40, 41, 47, 48, 52, 53, 57, 59, 60].

**Table 1:** Assessment of methodological quality for 23 studies retrieved examining KJLs in cutting tasks

Question	Criteria	Chaudhari et al. [55]	Cortes et al. [47]	David et al. [56]	David et al. [57]	Dempsey et al. [48]	Dempsey et al. [31]	Donnelly et al. [49]	Fedie et al. [59]	Havens et al. [34]	Jamison et al. [40]	Jamison et al. [35]	Jamison et al. [50]	Jones et al. [36]	Jones et al. [58]	Kristianslund et al. [61]	McBurnie et al. [60]	McLean et al. [51]	Mornieux et al. [52]	Sigward et al. [53]	Sigward et al. [37]	Vanrenterghem et al. [38]	Weir et al. [54]	Weltin et al. [41]
1	Power analysis was performed and justification of study sample size	0	2	0	0	2	2	0	2	2	2	0	2	0	0	0	2	0	2	0	0	0	0	2
2	Athlete demographics were clearly defined: gender, age, body height, and body mass at time of test	2	2	0	2	2	2	2	2	2	2	2	2	2	2	2	2	2	2	2	2	2	2	2
3	Athlete characteristics were clearly defined: sport, experience or activity level and level of play	2	1	0	2	1	2	2	2	2	2	1	1	1	1	1	2	2	2	1	2	1	1	2
4	Inclusion and exclusion criteria were clearly stated for athletes	2	1	0	0	0	0	0	1	1	2	0	1	0	0	0	2	1	0	1	2	0	0	2
5	Proper training and practice trials of the test were given to the athletes allowing for adequate familiarisation	1	2	1	2	2	2	1	2	0	0	0	0	2	2	2	2	0	1	0	2	2	0	2
6	Methods were described in great detail to allow replication of the test. Testing devices, *n* of trials, *n* and duration of rest, speed, angle of COD	1	2	2	2	2	2	2	2	2	1	1	1	2	2	2	2	2	2	2	2	2	2	1
7	Test-retest reliability of measurement device reported	0	0	0	0	0	0	0	0	0	0	0	0	0	0	0	0	0	0	2	0	0	1	0
8	Outcome variables clearly defined	2	2	2	2	2	2	2	2	2	2	1	1	2	2	2	2	2	2	2	2	2	2	2
9	Statistical analyses were appropriate	1	2	2	2	2	2	2	2	2	2	1	2	2	2	2	2	2	1	2	2	2	2	2
	Total score (maximum 18)	11	14	7	12	13	14	11	15	13	13	6	10	11	11	11	16	11	12	12	14	11	10	15

### Trunk

Eleven studies [31, 35, 36, 40, 41, 48, 50, 52, 54, 55, 61] identified trunk biomechanics as a biomechanical determinant responsible for generating large KJLs namely KAMs, or targeted the trunk in an intervention with the view to reducing KJLs and are presented in Table 2. Being the largest segment of the body, containing approximately half of the body’s mass, trunk positioning plays a significant role regarding the direction of the GRF vector during the weight acceptance phase of the cut. A contralateral lean of the trunk in the opposite direction of travel results in a greater laterally directed GRF [36]. This results in a greater perpendicular distance between the GRF and the knee, and increases KJLs due to a greater moment arm of the GRF in the frontal plane. Numerous other studies retrieved identified lateral trunk flexion as being a significant contributor to KJLs in pre-planned [36, 48, 55, 61] and unplanned [35, 52, 54] cuts of varying magnitudes (Table 2).

**Table 2:** Studies whereby trunk motion has observed to be contributory to KJLs

Study	Participants	Cuts used	Findings
Chaudhari et al., 2005 [55]	11 healthy subjects (6 F, 5 M)	4 m approach 90° PP cut unconstrained, and three sport-specific conditions including holding a lacrosse stick and football in the cutting side arm, and a football in the non-cutting side arm.	Constraining plant side arm with lacrosse stick and football result in ↑KAM of 60% and 29% respectively (*p* = 0.03)
Dempsey et al., 2007 [48]	15 M healthy team sports athletes	PP 45°cut modifying different technical parameters such as foot-plant distance, contralateral trunk lean and foot progression angle	↑ Foot-plant distance and contralateral trunk lean resulted in ↑ KAMs (*p* < 0.0001 and 0.030 respectively)
Dempsey et al., 2009 [31]	12 M healthy team sports athletes	PP and UP 45° cuts	6 weeks technique modification significantly ↓ in FP distance (*p* = 0.039 PP (ES = 0.55), UP (ES = 0.5)) and torso LF (*p* = .005 PP (ES = 1.09), UP (ES = 0.14)) leading to 36% ↓ in KAMs (*p* = 0.034; PP (ES = 0.58), UP (ES = 0.78)). Both postural changes were correlated with the change in KAM: FP distance ( *r* = -0.468, *p* = 0.025), LTF (*r* = − 0.377, *p* = 0.135)
Jamison et al., 2012 [35]	29 (15 M, 14 F) healthy subjects	Three steps at a self-selected pace UP 45° cut	Torso angle (outside tilt = (*p* = 0.02)) and torso GRF shoulder angle (*p* = 0.036) ↑ KAMs.
Jamison et al., 2012 [40]	36 M high school American footballers	Three steps at a self-selected pace UP 45° cut	6-week TS programme elicited no reduction in KAMs (*p* = 0.116)
Jamison et al., 2013 [50]	46 (23 M, 23 F) healthy subjects	Three steps at a self-selected pace UP 45° cut	All co-contraction indices and avg%diff of IO EO and L5 not significantly associated with KAMs or contralateral trunk lean *p* = 0.741, 0.782 and 0.233 for KAMs and *p* = 0.419, 0.947 and 0.439 for LTF.
Jones et al., 2015 [36]	26 elite and sub-elite F footballers	10 m approach 3 m exit of PP 90° cut	LTF sig correlated to KAM (*R* = − .42, *R*^2^ = 18; *p* = 0.05)
Kristianslund et al., 2014 [61]	123 F handball players	Handball-specific protocol—self-selected cut when receiving a ball and cutting around a static defender (mean cutting angle = 67°)	1 SD (8.6°) ↑ in LTF results in 7% ↑ KAM LTF significant predictor of pKAM (*ß* = 0.0090, *p* < 0.001) and moment arm of GRF at time of pKAM (*ß* = 0.00032; *p* < 0.001)
Mornieux et al., 201 [52]	13 M amateur footballers	4 m approach PP and UP 45 cuts 850-, 600- and 500-ms delays in stimulus presentation for UP cuts	LTF and KAM ↑ with reduced preparation time PP and 800 ms vs 500 ms (*p* = 0.05) LTF sig correlated with KAM (*r* = 0.41; *p* = 0.009)
Weir et al., 2019 [54]	30 F hockey players (15 junior, 15 elite)	UP 45° cutting task	Linear regression revealed that LTF and TF sig predictors of KAM (*p* = 0.05)
Weltin et al., 2017 [41]	28 F elite and sub-elite team sports athletes	UP 45° cutting task	PPT ↓CTR (*p* = 0.008, *η*^2^ = 0.277), step width (*p* = 0.029, *η*^2^ = 0.199) and ↑ pelvic axial rotation (*p* = 0.049, *η*^2^ = 0.165). No ↓ in KAMs (*p* = 0.605)

*Avg%diff* average percentage difference, *CTR* contralateral trunk rotation, *TF* trunk flexion, *TS* trunk stabilisation, *IO* internal obliques, *EO* external obliques, *KAM* knee abduction moment, *L5* L5 extensors, *LTF* lateral trunk flexion, *FP* foot plant, *ms* milliseconds, *PPT* perturbed plyometric training, *η*^*2*^ partial eta squared, *PP* pre-planned, *UP* unplanned, *SD* standard deviation, *M* male, *F* female, *ROM* range of motion, *ß* beta value, ↑ increased, ↓ decreased, *ES* effect size

Three interventions [31, 40, 41] targeted the trunk with regard to lowering KJLs in unplanned cuts [40, 41] and alongside pre-planned cuts of 45° [31]. Of these, two observed no changes in KAMs as a result of an intervention of static trunk exercises [40], and perturbed plyometric training [41] respectively. Dempsey et al. [31] elicited a meaningful reduction in KAMs following a technique modification protocol of reducing trunk lean and foot-plant width (Table 2).

### Hip

Eleven studies have identified hip kinematics or kinetics as determinants in generating greater KJLs in cutting tasks (Table 3). Of these, 2 identified sagittal plane kinematics/kinetics as determinants of KAMs [51, 60] and IRMs [60]. Seven identified frontal plane mechanics as determinants of KAMs [31, 36, 48, 53, 54, 59, 61] and IRMs [54, 60]. Finally, 5 studies [34, 37, 51, 53, 61], implicated transverse plane motion such as hip internal rotation as a determinant of KAMs.

**Table 3:** Studies identifying hip kinematics and kinetics in generating large KJLs.

Study	Participants	Cuts used	Findings
Dempsey et al., 2007 [48]	15 M healthy team sports athletes	PP 45° cut modifying different technical parameters such as foot-plant distance, contralateral trunk lean and foot progression angle	↑ Foot-plant distance and contralateral trunk lean resulted in ↑ KAMs (*p* < 0.001 and 0.030 respectively)
Dempsey et al., 2009 [31]	12 M healthy team sports athletes	PP and UP 45° cuts	6-week technique modification significantly ↓ in FP distance (*p* = .039) and torso LF (*p* = .005) leading to 36% ↓ in KAMs (*p* = 0.034)
Fedie et al., 2010 [59]	38 (19 M, 19 F) NCAA Div. 3 basketball players	Basketball-specific cutting protocol 35°–60°. PP cut or UP cut consisting of possibly receiving a basketball pass	↑Hip ABD (*p* = 0.02) in UP conditions alongside ↑ KADM in UP conditions (*p* = 0.032)
Havens et al., 2015 [34]	25 (12 F, 13 M) healthy DIv1-3 soccer players	45° and 90° PP cuts with 7.5 m approach and 7.5 m exit	↓ Hip INT ROT = ↑ KAMs (*R*^2^ = 0.25; *p* = 0.005) in 90° cuts
Jones et al., 2015 [36]	26 elite and sub-elite F footballers	10 m approach 3 m exit of 90° cut	↑ LLPD sig predictor of KAM (*R* = .45, *p* = 0.05; *R*^2^ = 20%)
Kristianslund et al., 2014 [61]	123 F handball players	Handball-specific protocol—self-selected cut when receiving a ball and cutting around a static defender mean 67°	Hip ABD sig predictor of pKAM (*ß* = 0.0201; *p* < 0.001) and moment arm of GRF at pKAM (0.00068; *p* = <0.001) Hip INT ROT sig predictor of pKAM (*ß* = 0.0111; *p* < 0.001)
McBurnie et al., 2019 [60]	34 elite and sub-elite M soccer players	70–90° PP cutting task with a 10 m approach and 3 m exit	PHFM sig predictor of KAM (*R* = − .624; *p* = < 0.001) and kIRM (*ρ* = 0.517; *p* = 0.002)
McLean et al., 2005 [51]	20 NCAA athletes (10 M, 10 F)	45° PP cutting task	↑Hip INT ROT predictive of KAM (*R*^*2*^ = 0.56—males, *R*^*2*^ = 0.60—females; *p* = 0.05) ↑Hip FLX predictive of KAM *(R*^*2*^ = 0.16—males, *R*^*2*^ = 0.19—female; *p* = 0.05)
Sigward et al., 2007 [53]	61 F soccer players	45° PP cutting task	High KAM group exhibited: ↑Hip ABD (*p* = 0.002, ES 0.79) ↑Hip INT ROT (*p* = 0.008, ES 0.71)
Sigward et al., 2015 [37]	45 (20 F, 25 M) healthy soccer players	45 and 110° UP cutting tasks with 7 m approach	vGRF, lGRF, Hip INT ROT and KAA = *R*^2^ 62.9% in 45° cuts (F_4,40_ = 19.654 , *p* < 0.001) in KAMS pGRF, Hip INT ROT and KAA = *R*^2^ = 41.5(F_3,41_ = 11.413, *p* < 0.001) in KAMs
Weir et al., 2019 [54]	30 F hockey players (15 junior, 15 elite)	UP 45° cutting task	Peak Hip ABD angle = sig independent predictor of KAMs (*ß* = 0.011, *p* = 0.046) Peak Hip ABD = sig independent predictor of kIRMs (*ß* = − 0.007; *p* = 0.002)

*INT ROT* internal rotation, *FLX* flexion, *PHFM* peak hip flexor moment, *Hip ABD* hip abduction, *KAA* knee abduction angle, *KAM* knee abduction moment, *pKAM* peak knee abduction moment, *KADM* internal knee adduction moment, *kIRM* knee internal rotation moment, *vGRF* vertical ground reaction force, *pGRF* posterior ground reaction force, *lGRF* lateral ground reaction force, *mlGRF* medio-lateral ground reaction force, *LLPD* lateral leg-plant distance, *PP* pre-planned, *UP* unplanned, *SD* standard deviation, *M* male, *F* female, *ß* beta value, ↑ increased, ↓ decreased, *R*^2^ coefficient of determination

Seven studies retrieved observed frontal plane hip motion, namely hip abduction to influence the magnitude of KAMs [31, 36, 48, 53, 54, 59, 63] and IRMs [54] in cuts of 45° [31, 48, 54], 90° [36], and self-selected ranges of 35–60° [59] and 70–90° [60] and a mean angle of 67° [61]. Excessive levels of hip abduction or lateral foot-plant distance (often used interchangeably) would result in the distal portion of the femur to be aligned medial to the GRF vector, resulting in greater KJLs. Greater abduction would also result in greater laterally orientated GRFs [36], resulting in a greater lever arm from the application of force relative to knee joint centre further amplifying KJLs.

Five studies identified transverse plane hip motion as a determinant of KJLs [34, 37, 51, 53, 61]. Four of the five studies identified an increased amount of internal hip rotation as responsible for generating greater KJLs [37, 51, 53, 61]. Transverse plane motion (internal rotation of the hip) would result in the knee being placed in a more medial position to the GRF vector and increase the perpendicular distance of the moment arm which would amplify the GRF resulting in a greater KAM. A significantly greater abundance of internal hip rotation (Table 3) was observed in a cohort of female athletes that generated greater KAMs than their counterparts, during 45° cuts [53]. Similar findings were observed whereby greater internal rotation of the hip is a determinant of KAMs in 45° [51], self-selected [61] and unanticipated 45° and 110° cuts [37]. Conversely, one study identified increased internal hip rotation as significantly associated with a reduction of KAMs in 90° cuts [34]. Finally, two studies identified sagittal plane biomechanics in the form of hip flexion and hip flexor moment as determinants of KAMs in 45° [51] and 70–90° [60] cuts.

### Knee

Several retrieved studies identified biomechanical variables about the knee as significant modulators of KJLs [34, 36, 37, 51, 54, 60, 61] (Table 4). Of these, two examined KJLs in pre-planned [51] and unplanned [54] 45° cuts. Two studies examined 45° and 90° pre-planned [34] and unplanned 45° and 110° cuts, respectively [37]. Two studies utilised a self-selected protocol of 35–60° [59] and a mean cutting angle of 67° [61]. Five [36, 37, 51, 54, 61] of the above studies identified frontal plane knee motion (knee abduction angle) as a determinant of KJLs. A knee valgus position is problematic, by placing the knee more medial to the GRF vector and amplifying the moment arm in the frontal plane resulting in a greater KJL. Two studies [34, 60] identified multiplanar moments of the knee in the form of internal knee extension moments (external KFMs), knee internal rotation moments (KIRMs) and knee flexion moments (KFMs) as responsible for eliciting greater KAMs.

**Table 4:** Studies implicating biomechanical variables about the knee in generating large KJLs

Study	Participants	Cuts used	Findings
Havens et al., 2015 [34]	25 (12 F, 13 M) healthy soccer players	45° and 90° PP cutting tasks with 7.5 m approach and 7.5 exit	KEM sig determinant of KAM (*R*^2^ change 0.17, *p* = 0.041)
Jones et al., 2015 [36]	26 elite and sub-elite F soccer players	10 m approach 3 m exit of PP 90° cut	Initial KAA sig predictor of KAM (*R* = − .67, *p* = 0.001; *R*^2^ = 45%)
Kristianslund et al., 2014 [61]	123 F handball players	Handball-specific protocol—self-selected PP cut when receiving a ball and cutting around a static defender mean 67°	KAA sig predictor of pKAM (*ß* = 0.704, *p* = <0.001), moment arm (*ß* = 0.00218, *p* = <0.001) and GRF at time of pKAM (*ß* = 0.212, *p* = .001)
McBurnie et al., 2019 [60]	34 elite and sub-elite M soccer players	70–90° PP cutting task with a 10 m approach and 3 m exit	Peak KFM and KRM sig determinants of KAM (*R* = 0.549 and − 0.488; *p* = 0.002 and 0.003 respectively)
McLean et al., 2005 [51]	20 NCAA athletes (10 M, 10 F)	45° PP cutting task	KAA predictive of KAM (*R*^*2*^ = 0.21—males, *R*^*2*^ = 0.35—females; *p* = 0.05)
Sigward et al., 2015 [37]	45 (20 F, 25 M) healthy soccer players	45 and 110° UP cutting tasks with 7 m approach	vGRF, lGRF, Hip INT ROT and KAA = *R*^2^ 62.9% in 45° cuts (F_4,40_ = 19.654, *p* < 0.001) in KAMs pGRF, Hip INT ROT and KAA (*R*^2^ = 41.5(F_3,41_ = 11.413, *p* < 0.001) in KAMs
Weir et al., 2019 [54]	30 F hockey players (15 junior, 15 elite)	UP 45° cutting task	KFA at IC sig predictor of KAM (*ß* = − 0.019, *p* = 0.001)

*INT ROT* internal rotation, *KAA* knee abduction angle, *KAM* knee abduction moment, *pKAM* peak knee abduction moment, *KFA* knee flexion angle, *KEM* knee extension moment, *KFM* knee flexion moment (external), *KRM* knee rotation moment, *IC* initial contact, *vGRF* vertical ground reaction force, *pGRF* posterior ground reaction force; *lGRF* lateral ground reaction force, *PP* pre-planned, *UP* unplanned, *M* male, *F* female, *ß* beta value, ↑ increased, ↓ decreased, *R*^2^ coefficient of determination, *sig* significantly

### Determinants of Multiplanar KJLs

Three studies examined the biomechanical determinants of multiplanar KJLs [35, 54, 60]. Of these, two utilised an unplanned 45° cut [35, 54], and the third adopting a pre-planned cut of 70–90° [60]. A range of biomechanical kinematics and kinetics were implicated in the generation of multiplanar knee loads and are presented in Table 5.

**Table 5:** Studies identifying determinants of multiplanar loads about the knee

Study	Participants	Cuts used	Findings
Jamison et al., 2012 [35]	29 (15 M, 14 F) healthy subjects	Three steps at a self-selected pace UP 45° cut	Contralateral trunk lean Sig negatively associated with pKIRM (slope = − 0.03, *p* = 0.021)
McBurnie et al., 2019 [60]	34 elite and sub-elite M soccer players	PP 70–90° cutting task with a 10-m approach and 3 m exit	Average ML GRF and average and peak hGRF sig determinants of pKRM (*R* = − 0.638, 0.581 and 0.576 respectively; *p* = < 0.001) pKAM and pKRM sig determinants of pKFM (*R* = − 0.549, − 0.494; *p* = 0.002 and 0.003 respectively).
Weir et al., 2019 [54]	30 F hockey players (15 junior, 15 elite)	UP 45° cutting task	KAA and KFA ROM sig independent predictor of pKEM (*ß* = 0.009, 0.010; *p* = 0.033 and 0.030 respectively. KAA, peak Hip ABD and KFA at IC sig independent predictors of pKIRM (*ß* = − 0.003, − 0.007, 0.002; *p* = 0.009, 0.002 and 0.280 respectively)

*Hip ABD* hip abduction, *KAA* knee abduction angle; *pKAM* peak knee abduction moment, *KFA* knee flexion angle, *KEM* knee extension moment, *pKFM* peak knee flexion moment, *pKRM* peak knee rotation moment, *pKIRM* peak knee internal rotation moment, *IC* initial contact, *PP* pre-planned, *UP* unplanned, *M* male, *F* female, *ß* beta value, *Sig* significantly, *ML* medio-lateral, *GRF* ground reaction force, *hGRF* horizontal ground reaction force, *R* Pearson’s correlation coefficient

### Ankle and Foot

Five studies [47, 49, 53, 56, 61] identified the ankle or foot as a determinant of KJLs. Three utilised 45° cuts, in unanticipated [47, 49] and pre-planned [53] conditions respectively. The other two consisted of pre-planned cutting tasks of 90° [56] and a self-selected protocol [61]. Of these, four [47, 49, 56, 61] studies identified sagittal plane alignment of the ankle as a significant determinant of KJLs. Three [49, 56, 61] of these studies identified a rearfoot strike as responsible for generating greater KAMs, with the remaining study observing opposite results to the contrary [47]. One study retrieved also identified transverse plane motion of the foot (a greater inverted foot) as a determinant of KAMs in an excessive “at-risk” group [53]. This would be due to the inverted foot placing the knee in a more compromised valgus position, for the foot to be orientated towards the direction of intended travel.

### Ground Reaction Forces, Penultimate Foot Contact and Braking Strategy

Seven studies (Table 7) identified ground reaction forces or braking strategy as determinants of KJLs. Of these, three utilised 90° pre-planned cuts [36, 57, 58], two pre-planned 45° cuts [38, 53], one study examined a pair of cuts of 45 and 110° [37]; the remaining study used a cut of 70–90° [60]. Four [36, 57, 58, 60] examined the kinematics and/or kinetics during the preceding penultimate foot contact (PFC) in order to measure the effectiveness of braking strategies, to which two identified PFC variables as determinants of KJLs [57, 58]. Finally, four studies [37, 38, 53, 58] identified multiplanar GRFs as determinants of KJLs.

## Discussion

The primary aims of this systematic review were to critically evaluate the current literature regarding the determinants of KJLs during cutting tasks, and use this in order to construct a technical framework in order to inform practitioners regarding the management of KJLs in cutting. In addition, it was also the aim to identify areas of further research, limitations within the literature and provide recommendations to practitioners concerning the management of athletes regarding KJLs in cutting tasks. Twenty-three articles retrieved directly examined the biomechanical determinants of KJLs in cutting tasks. Of these, 11 implicated trunk motion as determinant of KJLs, 11 identifying hip biomechanics, seven about the knee, three examining multiplanar KJLs, five implicating foot and/ or ankle positioning, and seven identifying GRFs and braking strategy as contributory factors regarding KJLs.

### Trunk

**Fig. 2:**
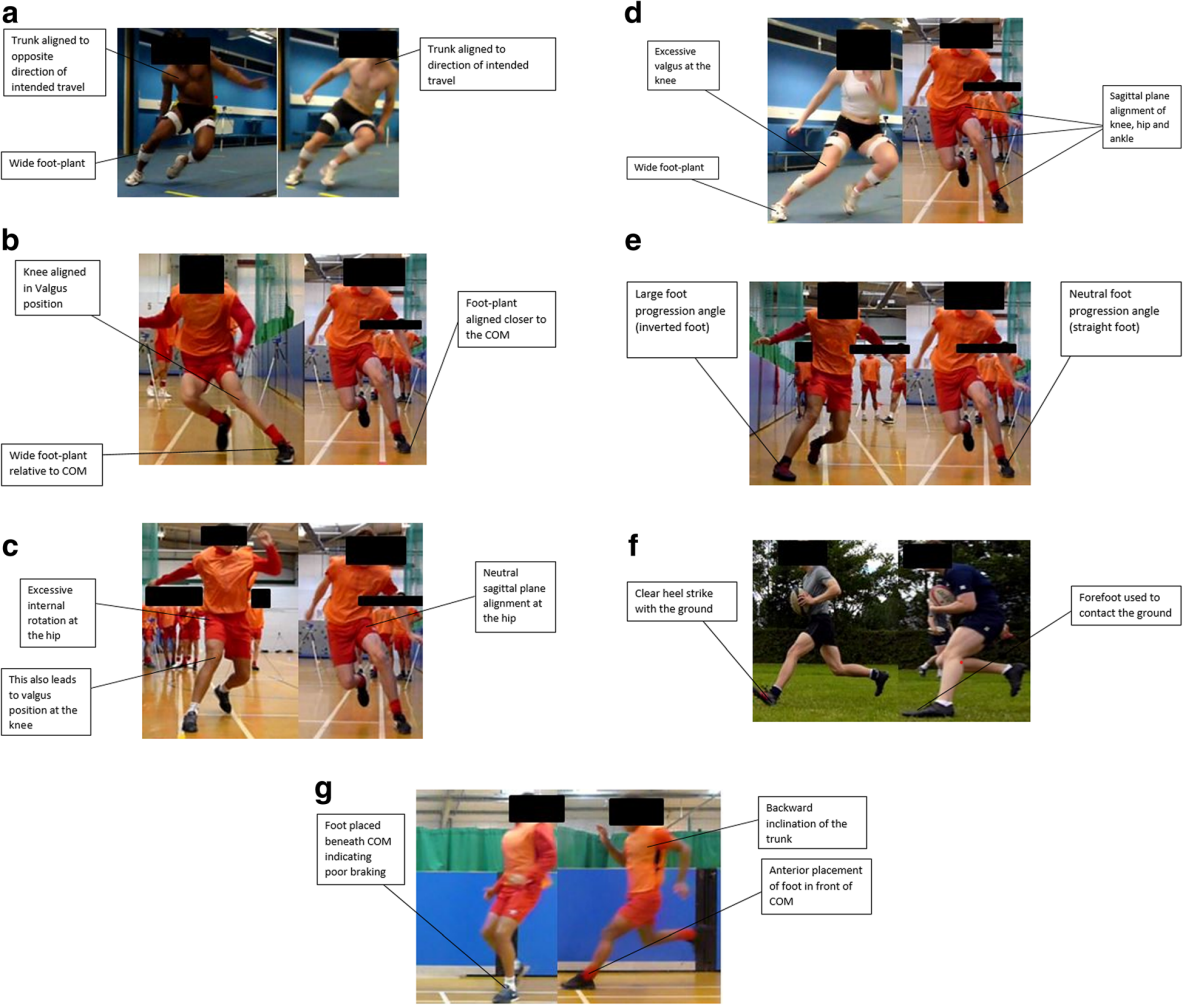
**a** Sub-optimal (left) and optimal (right) trunk alignment in cutting. **b** Sub-optimal (left) and optimal (right) frontal plane hip alignment in cutting. **c** Sub-optimal (left) and optimal (right) transverse plane hip alignment in cutting. **d** Sub-optimal (left) and optimal (right) frontal plane knee alignment in cutting. **e** Sub-optimal (left) and optimal (right) foot progression angles in cutting. **f** Sub-optimal (left) and optimal (right) foot landings in cutting. **g** Sub-optimal (left) and optimal (right) PFC braking in cutting

Trunk positioning is integral to mitigating KJLs during cutting (Table 2), due to the effect upon the GRF orientation and frontal plane moment arm of the knee as described above. Optimal trunk positioning therefore would constitute of the athlete leaning towards the intended direction of travel to mitigate this risk. High-risk and optimal trunk positioning are presented in Fig. 2a respectively.

Trunk positioning also has specific implications for athletes in ball carrying or implement carrying sports such as rugby, American football, lacrosse and hurling whereby the carrying of an object could constrain the plant side arm, and result in increased contralateral trunk flexion or possible rotation. Chaudhari et al. [55] identified that constraining the plant side arm by the trunk in numerous sport-specific conditions such as holding a football and lacrosse stick resulted in significant increases in KAMs, against a non-constrained control condition (Table 2). This is of importance for practitioners involved in ball or implement carrying sports, as there appears to be a propensity to generate greater KAMs due to the influence that carrying an object can have on frontal plane motion. Although carrying a ball or implement can increase KJLs, athletes cannot simply put down their stick or refuse to carry the ball if required. Therefore, prioritising sagittal plane alignment of the lower limb and training appropriate deceleration technique is suggested as a more effective way of reducing KJLs in these populations, due to the demands of implement carrying sports.

If certain sub-optimal movement patterns have succinct relationships with contraindicative knee loading, it is of interest to further understand what actually causes poor trunk alignment, so practitioners can appropriately address whether it is a technique deficit, or one on a neuromuscular level. Jamison et al. [50] examined pre-activation in the core muscles via percent differences and co-contraction indices, and hypothesised that possible asymmetry in activation could be responsible for a trunk misalignment, and therefore KAMs. No significant differences were found across a multitude of asymmetry co-contraction index differences and KAMs, and lateral trunk lean (Table 2) suggesting that poor trunk control in cutting is not due to a neuromuscular trunk deficit. Therefore, technique modification may be more appropriate in addressing trunk lean as opposed to strengthening trunk musculature.

Numerous interventions have attempted to address trunk movement in order to reduce KJLs in cutting with mixed success [31, 40, 41]. Considering that greater KJLs are caused by numerous segmental contributions, adopting a multi-segmental approach in intervention may be most effective in eliciting reductions. Dempsey et al. [31] adopted a technique modification approach whereby a reduction in trunk lateral flexion with a reduced foot-plant width elicited a 36% reduction (Table 2) in KAMs in 12 athletes over a 6-week intervention. Additionally, change in torso lateral flexion exhibited a moderate relationship (*r* = − .377) with KAM. However, the failure to include a control group and to report performance times pre-post intervention is worth noting, as athletes will seldom engage in a programme that will be detrimental to performance [34]. Jamison et al. [40] failed to elicit any reduction in trunk lean or KAMs in 11 males following 6 weeks of a trunk stabilisation programme that incorporated prone planks, side planks and other trunk stabilisation exercises, in unanticipated 45° cuts. A shortcoming of the study to only utilise static stabilisation exercises may explain these findings, as they would have limited transfer to high velocity dynamic movements such as cutting.

More dynamic training methods such as plyometrics may carry greater efficacy in modifying KJLs during cutting tasks. This is resultant of the need to attenuate and redirect multiplanar GRFs, which would carry improved transfer into cutting. Using novel methods, Weltin et al [41] examined the utility of perturbed and unperturbed plyometric training in reducing KAMs. Significant reductions were observed in contralateral trunk and pelvic rotations (Table 2) although this did not translate into a reduction in KAMs. However, increased levels of pelvic axial rotation were present, which could indicate a change in strategy or a preparatory movement prior to cutting. Increased pelvic motion in the transverse plane would result in a more medially placed limb relative to the GRF that increases KAMs [36, 51, 54, 61] and could have offset any potential KJL reduction from corrected trunk motion. Future research examining the interaction between preparatory strategies, different body segments and their respective contribution to KJLs is recommended. A greater understanding of these preparatory strategies would be useful for practitioners and could inform technique-orientated interventions.

Athletes should aim to align the trunk to the intended direction of travel, in order to reduce KJLs. Examining the effectiveness of a multi-segmental approach when aiming to reduce KJLs is warranted, considering the only study to elicit a meaningful reduction [31] adopted this strategy. This should be tested through more interventions carried out in “high-risk” populations utilising more dynamic exercises and technique modification, as opposed to the utilisation of static exercises. Following this, any effect on performance could be observed and possibly inform future practice and compliance if said interventions had a positive effect on performance times alongside ameliorating the magnitude of KJLs.

### Hip

Frontal and transverse plane hip alignment is imperative when attempting to mitigate KJLs during cutting (Table 3). Malalignment of the hip in either of these planes can result in sub-optimal orientation of the distal joints (knee and ankle) further down the kinetic chain. This results in a greater frontal plane moment arm about the knee, due to a medially positioned knee relative to the GRF and laterally directed GRFs [36]. Therefore, when cutting, athletes should avoid excessive abduction and internal rotation of the hip to mitigate KJLs. Figure 2 b and c display sub-optimal and optimal hip alignment in the frontal and transverse planes respectively.

The need to attenuate and redirect forces in the sagittal plane is of great importance when changing direction, considering the mechanisms of frontal and transverse hip alignment that amplify KJLs. Although limited evidence has implicated sagittal plane biomechanics as a determinant of KJLs. McLean et al. [51] elucidated increased hip flexion to be contributory to greater KAMs in 20 NCAA athletes. This is somewhat counterintuitive when considering the relationships that frontal and transverse plane hip motions share with KAMs [37, 53, 61]. Despite the numerous studies retrieved, this finding has not been substantiated elsewhere. This could however be attributed to the low percentage of variance explained in KAMs by hip flexion (*R*^2^ = 16%), despite the significance of the findings.

McBurnie et al. [60] identified peak hip flexor moments as a strong correlate of KAMs and IRMs (Table 3) in 70°–90° cuts, a somewhat novel finding. Peak hip flexor moments have been suggested as having a protective role against KAMs in 180° pivots [64]. Greater sagittal plane alignment would allow for better absorption of loads through large musculature of the thigh and hip, and thus reduce loading through utilising the substantial eccentric strength of these muscle groups. However, the lower reduction in velocity present throughout a cutting movement due to a desire to maintain velocity along the path of the cut could explain this finding. This would amplify the GRF generated [38] and thus greater moments about the hip, eliciting a greater knee joint load. Considering the above, alongside sagittal plane hip power and extensor moment being identified as determinants of 45° cutting performance [34], it is possible that the biomechanics required for faster cutting performance may be disparate with those for mitigating KJLs and injury risk as previously proposed [34, 60].

First identified by Havens [34], it appears there is a clear conflict between optimal cutting performance biomechanics, and those optimal for mitigating KJLs. Faster completion times require shorter ground contact times [65] and a greater approach velocity, rate of deceleration and GRFs to be produced [60, 66]. This would also result in a greater GRF [38] being amplified by any frontal plane moment arm, which would result in greater KJLs. Greater hip flexor moments previously were identified as a significant determinant of KAMs and performance time in 70–90° cuts [60], and identified as having a strong relationship with 45° cut completion time [34]. In addition, medio-lateral separation distance has also been identified as a strong predictor of both cutting performance and KJLs [34]. Furthermore, McBurnie et al. identified KAMs, IRMs and KFMs (external) to possess significant relationships with faster cutting performance times (*R* = − 0.590; *d* = − 1.0; *R* = 0.525; *p* = 0.001; *R* = − 0.509; *p* = 0.002 respectively) and a presence of greater knee abduction angles in faster performers in a 70–90 cut°. Taken together, the greater kinetics and moments that are required for performance will undoubtedly elevate KJLs. However, the detrimental effect of these despite their need could be mitigated through minimising the frontal plane moment arm to which these forces can be amplified. In addition, it is proposed that effective deceleration training could reduce the magnitude of the GRF in the final contact, where injury commonly occurs.

There is strong evidence for constraining frontal plane hip motion during cutting tasks, due to the effect of increasing the frontal plane lever arm which amplifies the GRF (Table 3). If one abducts the hip to a greater extent, it would shift the centre of mass (COM) and GRF laterally, possibly causing a greater contralateral trunk lean, which as discussed above would elicit greater KAMs. This combination has been previously observed [54] and is further consolidated by Dempsey et al.’s [31] intervention study whereby reducing these two parameters resulted in a significant reduction in KAMs (36%, *p* = 0.034). This multi-segmental approach is recommended to practitioners to maximise intervention effectiveness, although further research is required to determine if a more medial foot plant would elicit performance detriment.

It appears there may be a greater requirement for a wider foot plant in cuts of a greater degree (90° and 67° respectively) [36, 61] in order to generate greater perpendicular forces, which may explain relationships of hip abduction with increased KJLs in these tasks. During unconstrained cuts, Kristianslund et al. [61] found hip abduction to be significantly contributory to KAMs (Table 3). More interestingly, the cutting angle was also found to be synonymous with greater KAMs. This does highlight a somewhat mechanical influence that is caused by the discrete demands of the task, which has been recently discussed in the literature [39]. In further support, Jones et al. [36] identified lateral leg-plant distance as a significant determinant (Table 3) of KAMs in 90° cuts. A moderate (*R* = .59) correlation was also observed between lateral leg-plant distance and medial GRFs, substantiating the notion that a wide foot plant is required to generate the GRFs necessary to facilitate direction change in greater angled turns. Therefore, if greater angled cuts are being performed, it may be necessary to adopt a wide foot plant to meet the demand of the task. In this instance, it is recommended KJLs are mitigated through ensuring appropriate trunk positioning, sagittal plane alignment of the lower limb and sufficient braking prior to the foot plant.

Transverse plane hip motion has been identified as a significant determinant of KJLs (Table 3). Similarly, it appears there may be an angular interaction regarding this variable, with the majority of studies identifying internal rotation of the hip as a significant determinant of KJLs in 45° cuts (Table 3). Conversely, one study [34] found a decreased amount of hip internal rotation to be contributory to KAMs. This is somewhat counterintuitive, considering the plethora of research and theory supporting the idea that internal rotation would increase KJLs. Such results were attributed to the cutting angle used being of a greater magnitude (90°). Although Sigward et al. [37] identified internal rotation of the hip to be a significant predictor of KAMs in 110° cuts. However, it is possible that there may have been an element of “pre-rotation” of the pelvis which has been observed in greater angled CODs, which could explain such findings, as it would reduce the need for a greater amount of internal hip rotation through axial pelvis rotation, although an omission of such measurement from these studies leaves this theory speculative.

It is recommended that frontal and transverse plane hip motion is limited when cutting due to the propensity to generate greater KJLs. During greater angled cuts (> 70°) where there is a need to adopt a wide foot plant, it is recommended that KJLs are mitigated through appropriate trunk lean towards the intended direction and sagittal plane alignment of the lower limb. This is likely to reduce KJLs and performance times, although may result in a “deceptive disadvantage” (whereby an opponent may find it easier to anticipate the movement), thus highlighting further conflict between the biomechanics required for optimal performance and mitigating KJLs. Sagittal plane alignment is recommended to reduce the moment arm that would amplify the greater GRFs associated with faster performance. Appropriate braking and deceleration strategies may be useful in reducing the magnitude of the GRF generated in the final foot contact, whereby the greater loads are generated and injury occurs.

### Knee

Frontal plane knee motion would have a direct effect on KJLs, namely KAMs, due to placing the knee in a more medial position to the resultant GRF vector. This is a fundamental component of the “dynamic valgus” position proposed by Hewett [30] whereby an abducted knee, adducted and internally rotated hip, alongside an everted ankle and pronation have been observed as an established injury risk for non-contact ACL injuries. Sagittal plane alignment of the knee during cutting is therefore encouraged. Figure 2d displays optimal and sub-optimal frontal plane knee alignment in cutting tasks.

In a seminal study, McLean et al. [51] examined the determinants of KAMs in a cohort of 20 NCAA athletes in 45° cuts identifying initial peak valgus position to be predictive in both males and females. However, it is questionable if the determinants found would extrapolate to greater angled cuts that are mechanically discrete [39] that have been found to occur in abundance in team sports [28]. In contrast, adopting a self-selected cutting protocol, Kristianslund et al. [61] found initial knee abduction angle to be a significant predictor of KAMs in cuts of an uncontrolled magnitude. A 4.4° increase in abduction angle was found to correspond to a 19% increase in KAM. Similarly, Jones et al. [36] found knee abduction angle to correlate strongly with KAMs (Table 4) during 90° cuts in a cohort of female footballers, consolidating that an abducted knee is a universal biomechanical determinant of KAMs across all angles.

Considering that an abducted knee is contraindicative regarding knee loads during cutting, appropriate injury prevention practice would be to avoid such “risky” posture to reduce the likelihood of generating loads that will strain and could ultimately rupture the ACL. This has been typical practice in injury mitigation programmes to eliminate any valgus/ abduction alignment of the knee and appears warranted. A limitation of the majority of identified studies is that they adopt controlled approach velocities to identify determinants of KJLs; however, this may not be sufficient enough to elicit loads similar to the magnitude generated in sport-specific scenarios. Further research is recommended with uncontrolled approach velocities or maximal effort to examine if these identified determinants of KJLs remain present under greater velocity conditions. From the above, eliminating frontal plane knee alignment in line with current injury prevention guidelines is advised to practitioners working with athletes in cutting sports.

#### Determinants of Multiplanar Loads of the Knee

Externally applied knee flexion moments (KFMs) and internal rotation moments (KIRMs) have been identified as prominent ACL stressors [18, 21]. A knee close to full extension would amplify the moment arm of the GRF perpendicular to the knee and subsequently increase the amount of anterior shear force (ASF) generated, resulting in greater ACL loading [18]. In a similar manner, a greater KIRM would result in greater tibial angular acceleration, consequently resulting in greater ACL strain [21]. Jamison et al. [35] identified contralateral trunk lean as possessing significant negative associations with KIRMs (Table 5). However, caution is advised, due to the poor methodological quality observed (33% of desired criteria) and abovementioned relationship of trunk lean with KAMs. Unsurprisingly, frontal plane kinetics have been found to significantly predict KIRMs in sub-elite athletes alongside average and peak horizontal GRFs [60]. KFMs have been implicated as a determinant of the range of motion the knee goes through during cutting manoeuvres. A reduced knee ROM has been proposed to be contraindicative [54] due to an increased sagittal plane moment arm. However, moving through a greater knee flexion ROM would result in a greater ground contact time, which possesses an inverse relationship with performance times [65].

It is worth noting that multiplanar KJLs were all found to have significant associations with performance time in the study of McBurnie et al. [60] Taken together, with the aforementioned relationship regarding KFMs, this further signifies the performance injury conflict between “safe” cutting biomechanics and ones required for faster performance. Further research is recommended examining this phenomenon to identify the true extent to which it exists, and exploring ways to reduce KJLs without a significant detriment to performance. It is recommended that a knee close to full extension in the plant phase of the cut is avoided, due to the established relationships with ASF, and ACL loading. Landing with a “soft knee” (a knee flexion angle > 15°) is therefore warranted, due to increased ACL strain associated with extended knee postures [67].

### Ankle and Foot

Foot positioning, whether this may be inwardly or outwardly rotated, first cited in the literature as a “foot progression angle” [53] has been identified as predictive of KJLs. It can be postulated that an inverted foot angle that is needed to facilitate rotation of the body in a new direction could result in the knee being placed in a somewhat medially compromised position, due to the impact of tibial internal rotation needed to inwardly rotate the distal segment and foot. Conversely, an outwardly rotated foot would lead to an increased susceptibility to eversion and pronation, which could also lead to knee abduction and tibial rotation, and thus ACL loading [68–70].

Sagittal plane ankle positioning can also have an effect on KJLs through the type of footfall strike adopted. Within running literature [71, 72], it has been established that runners utilising a forefoot-strike pattern (i.e. plantarflexed) will generate significantly lower GRFs than rear-footfall pattern runners. Striking with the rearfoot and a knee joint close to extension would render the gastrocnemius complex passively insufficient, and thus inhibit their ability to attenuate GRFs, resulting in greater KJLs [4]. Therefore, it appears a relationship between the ankle and the knee whereby work and load absorption of the two joints are dependent on the footfall pattern adopted. Whether this could be advantageous for practitioners in managing knee joint loading in athletic populations requires further examination. Figure 2e exhibits sub-optimal and optimal foot progression angles. In addition, Fig. 2f exhibits sub-optimal and optimal landings respectively during cutting manoeuvres.

Conflicting results have been observed in the literature regarding foot progression angle [36, 53, 61]. Sigward and Powers [53] found a significant correlation between an inwardly rotated foot contact position to KAM (Table 6), although Jones et al. [36] found no relationship between foot progression angle when examining determinants of KAMs in 90° cuts. However, the “high-risk” participants in this study (exhibiting KAMs + 0.5 SD above the mean) had a substantially greater inward foot rotation compared to the low-risk cohort (0.5 SD below the mean) (Table 6), suggesting this relationship still exists. Such disagreement between results could be explained by differing cutting angles (45° vs 90°) and approach velocities (5.5–7.0 vs 4.0–5.0 m s^-1^). On the contrary, Kristianslund et al. [61] found no significant relationships between foot progression angle and KAMs. However, the use of a self-selected cut and inclusion of a static defender may provide some mitigation for these results, considering the pronounced effect on kinematics and kinetics that static defenders have been found to elicit [59].

**Table 6:** Studies identifying foot and ankle positioning as contributory to KJLs

Study	Participants	Cuts used	Findings
Cortes et al., 2012 [47]	20 F soccer Division 1 athletes	UP 45° task	RF landing = ↓ KADM in 45° cut (F _(1,18)_ = 11.882; *p* = 0.003)
David et al., 2017 [56]	50 participants (23 M, 27 F)	90° PP cutting task with 3 m approach	Habitual RF landing exhibited ↑ pKAM 11–19% of stance phase (*p* = 0.008)
Donnelly et al., 2017 [49]	19 elite F hockey players	45° UP cutting task	Habitual RF possess sig ↑ pKAM to FF (1.4 ± 0.5 Nm kg^−1^ vs. 0.5 ± 0.4 Nm kg^−1^; *p* = 0.001) Habitual FF = sig ↓ power absorption at knee (− 32.0 ± 7.5 W kg^−1^ vs −68.8 ± 18.5 W kg^−1^; *p* = < 0.001) and sig ↑ at ankle (− 15.3 ± 4.4 vs. − 5.8 ± 1.8 W kg^−1^; *p* = < 0.001)
Jones et al., 2015 [36]	26 elite and sub-elite F soccer players	10 m approach 3 m exit of PP 90° cut	“High-risk” group (exhibiting KAMs + 0.5 SD above the mean) had a substantially greater inward foot rotation compared to the low-risk cohort (0.5 SD below the mean) (14.7 ± 0.9° vs. 5.5 ± 1.2° respectively).
Kristianslund et al., 2014 [61]	123 F handball players	Handball-specific protocol—self-selected PP cut when receiving a ball and cutting around a static defender mean 67°	1SD (16°) increase in plantarflexion/toe landing corresponds to approximately a 13% decrease in KAM
Sigward et al., 2007 [53]	61 F soccer players	45° PP cutting task	↑ KAM group exhibit sig ↑ foot progression angle (*p* = 0.04, ES = 0.55). Foot progression angle sig correlated to pKAM (*R* = 0.39, *p* = < 0.001)

*FF* forefoot, *RF* rearfoot, *KAM* knee abduction moment, *KADM* internal knee adduction moment, *Nm kg*^*−1*^ Newton metres per second, *W kg*^*−1*^ Watts per kilogramme per second, *IC* initial contact, *PP* pre-planned, *UP* unplanned, *M* male, *F* female, *ß* beta value, ↑ increased, ↓ decreased, *R*^2^ coefficient of determination, *sig* significantly

Regarding sagittal plane ankle position, an increased downward landing angle (e.g. more plantarflexed/ toe landing) was also found to modulate KAMs, with a 16° increase in plantarflexion corresponding to approximately a 13% decrease in KAM [61]. Cortes et al. [47] found heel striking to produce lower KAMs compared to forefoot striking in unanticipated 45° turns, a somewhat counterintuitive finding. However, a lack of familiarisation and appropriate training (three practice trials), coupled with instructed technique suggests that this protocol does not represent individual movement strategies. Alongside this, there was no calculation of mechanical work done by each joint, failing to give a valid representation of the loads the ankle and knee had attenuated respectively.

Grouping athletes by their habitual foot-strike pattern would be more useful, as this would give a more accurate representation of their pre-learned motor programmes which would be used in game situations. Donnelly et al. [49] retrospectively identified habitual forefoot and rearfoot strikers in 19 elite female hockey players performing 45° cuts. Habitual forefoot strikers exhibited significantly lower KAMs alongside significantly lower power absorption at the knee (Table 6). Interestingly, this shared a somewhat inverse relationship with ankle power, whereby forefoot strikers absorbed significantly more power through the ankle (Table 6). These results show clear interactions between the knee and the ankle in terms of power absorption and load distribution. Further research is required to investigate interactions between segments and preferential load distribution further. David et al. [56] identified three habitual movement patterns in a cohort of 50 participants; a rearfoot strike, a forefoot strike with a clear impact peak and a “true” forefoot strike with no impact peak suggested to be resultant of pre-orientation of the body. Rearfoot striking resulted in greater KJLs than both habitual forefoot striking conditions (Table 6). Within the forefoot conditions, a pre-rotated trunk and backwards leaning resulted in lower knee loading than the other forefoot striking condition that was deemed to generate a KAM similar to that of a heel strike with an impact peak. This again emphasises the multi-segmental mechanism to which KJLs are generated.

From the above, it is recommended that a neutral foot progression angle is adopted when cutting to prevent any knee abduction or internal rotation of the tibia, which would elevate KJLs. Modification of foot-strike pattern may be useful, whereby adopting a more forefoot orientated “ankle dominant” strategy when cutting will reduce load and power absorption through the knee. This could have implications for the ankle joint, as forefoot striking has been reported to elicit considerable strain [73] and eccentric load [74] on the triceps surae muscle- tendon complex when running. During cutting, this would be amplified further by the large deceleration component present [33] and require substantial force production from the triceps to meet the demands of the task. Without sufficient strength present, this would elevate the risk of acute muscle strain injury. Foot-strike modification is not something yet to be observed in injury prevention programmes and further research is recommended to investigate if this would be beneficial in reducing knee joint loads in athletic populations without overloading the ankle joint.

### GRF, Penultimate Foot Contact and Braking Strategy

Despite the numerous biomechanical determinants that have been mentioned above, and their influential role in the modulation of KJLs, it must be noticed that these predominantly affect the lever arm of the moment. This either shifts the GRF orientation or knee position respective to such, resulting in a more medial, compromised dynamic valgus position of the knee relative to the GRF. Although the lever arm to which a moment operates about is significantly contributory to the resultant moment, it is also noteworthy that the orientation and magnitude of the GRFs in the penultimate and turning steps can influence KJLs.

In theory, a greater GRF that is generated during the foot contact initiating the turn would result in a greater KJL, due to the magnitude of the force generated, subsequently amplified by the respective moment arm. Greater GRFs have been observed in greater angled cuts (> 45°) [37] and in cuts performed at greater velocities [38]. In theory, if the magnitude of GRF was lower during the final contact, the KJL generated would be substantially lower. If one were to dissipate the majority of momentum in the steps prior to turning through applying greater antero-posterior braking forces, there would be a reduction in velocity (deceleration) through the impulse-momentum relationship (Impulse = Δ Momentum), which would result in a reduced GRF generated during the turning step. This could be accomplished by a “large anterior placement of the foot relative to the COM and backward inclination of the trunk relative to planted foot” [58] in the preceding steps. Figure 2g exhibits sub-optimal and optimal braking strategy during cutting tasks.

Sigward et al. [53] identified significantly greater laterally directed GRFs being up to three times greater (Table 7) in a “high-risk” increased KAM group, in line with other findings [36]. This was attributed to contacting the ground differently with a more medial orientation of the foot (resulting in greater lateral forces due to Newton’s third law) and may explain the strong relationship identified with an increased foot progression angle being a determinant of greater KAMs (*p* = 0.04). Instead of creating laterally directed GRFs as means to complete the task, it may be more advisable for practitioners to encourage greater amounts of pre-orientation as elucidated by David et al. [57]. A greater amount of pelvic rotation and so called “pre-orientation” resulted in a more ankle dominant loading pattern in terms of power absorption and joint work done. This could also be facilitated during the penultimate foot contact (PFC) to reduce the re-directional requirements during the final foot contact (FFC). Whether such a strategy modulates KJLs remains to be seen, and further research is recommended to investigate this theory.

**Table 7:** Studies identifying GRFs, penultimate foot contact and braking strategy as contributory to KJLS

Study	Participants	Cuts used	Findings
David et al., 2018 [57]	67 healthy participants (35 M, 32 F)	90° PP cutting manoeuvre	EARLY pre-orientation = ↓ PEN step width and ↑ pelvis rotation leading to FF strike pattern and sig ↑ load absorbed through the ankle as opposed to knee (43.8% vs 32.5% and 35% vs 40% respectively)
Jones et al., 2015 [36]	26 elite and sub-elite F soccer players	10 m approach 3 m exit of PP 90° cut	pHBFR exhibits no relationship to KAM (*R* = .03, *R*^2^ = < 1%) LLPD = moderately correlated with KAM (*R* = .59) mGRF sig correlated to LLPD (*R* = .45, *p* = 0.05; *R*^2^ = 20%) “High risk” (exhibiting KAMs + 0.5 SD above the mean) had a substantially greater inward foot rotation compared to the low-risk cohort (0.5 SD below the mean) (14.7 ± 0.9° vs. 5.5 ± 1.2° respectively).
Jones et al., 2016 [58]	22 elite F soccer players	PP 10 m approach 90° cutting task with 5 m exit	Average hGRF during PEN sig related to KAM in cutting (*R* = − 0.569, *R*^2^ = 32%, *P* = 0.006)
McBurnie et al., 2019 [60]	34 elite and sub-elite M soccer players	PP 70–90° cutting task with a 10-m approach and 3 m exit	No sig relationships observed between any PEN hGRF variables. Moderate effect size (*d* = 0.9, *p* = 0.05) for average hGRF in PEN between fast and slow performers Horizontal approach velocity sig moderately correlated to KAM (*R* = 0.414, *p* = 0.015)
Sigward et al., 2007 [53]	61 F soccer players	45° PP cutting task	Sig ↑ lGRF in “excessive valgus” group (1.5 ± 0.9 vs 0.4 ± 0.5 N/Kg BW; *p* = < 0.001)
Sigward et al., 2015 [37]	45 (20 F, 25 M) healthy soccer players	45 and 110° UP cutting tasks with 7 m approach	vGRF = *R*^2^ = 37%, *R* = 0.607, *p* = < 0.001 of KAMs in 45° cuts pGRF = *R*^2^ = 19%, *R* = 0.460, *p* = 0.001 of KAMs in 110° cuts
Vanrenterghem et al., 2012 [38]	14 healthy F athletes	45° PP cutting task at incrementally increasing velocities 2 m s^−1^ 3 m s^−1^ 4 m s^−1^ 5 m s^−1^	Increased approach velocity = sig ↑ KAMs (*p* = 0.05), sig ↑ pGRFs (*p* = 0.0005), sig ↑ mGRFs (*p* = 0.0005)

*FF* forefoot, *RF* rearfoot, *pKFM* peak knee flexor moment, *KAM* peak knee abduction moment, *vGRF* vertical ground reaction force, *pGRF* posterior ground reaction force, *hGRF* horizontal ground reaction force, *mGRF* medial ground reaction force, *LLPD* lateral leg-plant distance, *lGRF* lateral ground reaction force, *pHBFR* peak horizontal braking force ratio, *PEN* penultimate, *IC* initial contact, *PP* pre-planned, *UP* unplanned, *M* male, *F* female, ↑ increased, ↓ decreased, *R*^2^ coefficient of determination, *sig* significantly, *m s*^*−1*^ metres per second

Jones et al. [36] observed a moderate relationship between medial GRF and lateral leg-plant distance (*R* = .59). This is likely due to the nature of the 90° cut used which encompasses a substantial redirection component in comparison with lower angled cuts [33]. When taking into account that lateral leg-plant distance was identified as a significant predictor of KAM (Table 7), it is possible that medial GRFs are contributory to the generation of greater KAMs that are typically seen in greater angled cuts [37, 75]. Vertical GRFs (vGRFs) have also exhibited established relationships with KAMs, due to the magnitude of the GRF being contributory to the resultant and final moment generated about the knee [37]. A greater vertical force component would be present in lower angled cuts [75] due to the absence of greater re-directional demands, and an ability to perform such a task with a greater approach velocity, and such an angle-velocity trade-off has been highlighted in a recent review [39].

GRFs applied in the steps prior to the final contact that initiates the direction change can be a determinant of KJLs. A rationale for examining the preceding foot contacts of a cut when taking into account typical contact times in cutting (0.319 ± 0.06 s [61]) has previously been identified [58], inferring deceleration occurs over a multitude of steps. This has been substantiated by research that has examined braking characteristics occurring in the preceding footsteps in cutting tasks [33, 36, 58, 60]. Havens and Sigward [33] subsequently found disproportionately greater braking present in 90° cuts compared to 45° cuts. Braking demands were accommodated across both foot contacts for the 45° cut, although the need to produce a greater medio-lateral impulse to meet the more mechanically challenging nature of the 90° cut required greater ground reaction impulse to be applied. This increased contact time in the final and braking impulse in the penultimate foot contact. This emphasises the importance of deceleration and braking in turns of a greater magnitude (≥ 60°). However, a limitation of the aforementioned study is that the relationship between braking and KAMs was not directly examined.

Jones et al. [58] investigated the potential efficacy of braking strategies whilst investigating the biomechanical determinants of cutting, through examining a ratio of peak horizontal (antero-posterior) GRFs between the penultimate and final foot contacts. No relationship was observed between this ratio and KAMs [36]. However, solely examining peak variables may not provide an in-depth analysis of the braking effect that occurs when dissipating momentum over a multitude of steps. The authors mentioned their failure to consider average horizontal braking force and impulse, which in turn would provide greater insight into braking in the penultimate step. A greater average horizontal braking force applied over the time of contact during the penultimate step would lead to a greater impulse applied to the ground, causing a reduction in forward momentum through the impulse-momentum relationship. Subsequently, average horizontal GRF during the penultimate contact (Table 7) explained large amounts of variance in cutting in a follow-up investigation [58]. Consequently, this provides a rationale for practitioners to encourage braking prior to the final contact to lessen the risk of generating high KJLs during cutting. Recently, McBurnie et al. [60] found no significant relationships between any penultimate foot contact variables including peak and average horizontal GRFs and ratios in a cohort of 33 sub-elite footballers. However, the shorter approach distances between the studies (5 m vs 10 m and 15 m) may substantiate such differences.

Little is known yet regarding the underpinning strength and biomechanical qualities that facilitate braking capacity. Furthermore, there has been little research examining the distinct differences in braking profiles across a range of cutting angles. Further research is recommended examining the effect of eccentric strength on braking capacity and profiles, and whether this could translate into a mitigation of KJLs in cutting tasks. Practitioners are advised to emphasise the importance of a neutral (straight) foot position when coaching cutting technique to their athletes. It also appears there is usefulness in examining braking strategies with view to reducing KJLs. This can be accomplished by emphasising braking in the steps prior to initiating the cut, through a large COM–COP distance, and backward lean of the trunk. Consequently, less momentum would have to be dissipated in the final contact prior to turning, reducing ground contact times and allowing the athlete to redirect and reaccelerate in a faster manner. It is therefore possible that interventions improving braking strategy may address the performance injury conflict previously identified [34] and that improving braking ability may also translate into enhanced performance, alongside ameliorating KJLs.

## Conclusions

**Fig. 3:**
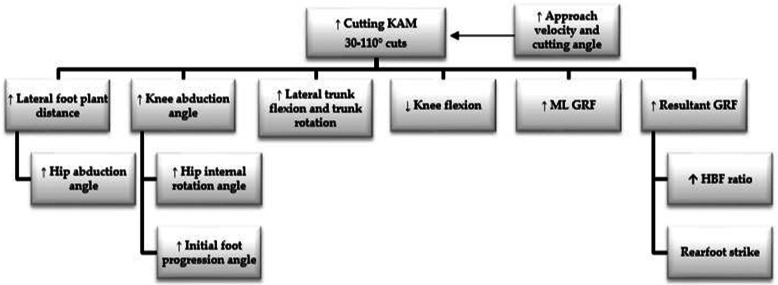
A deterministic model identifying the variables which amplify KAMs during cutting 30°–110°

Based on the associative work regarding determinants of KJLs during cutting (“Discussion” section), a deterministic model has been created identifying the variables which amplify KAMs during 30°–110° cutting, potentially predisposing athletes to an increased risk of non-contact ACL injury (Fig. 3). It should be noted that insufficient evidence is available at the moment to provide technical models for CODs between 110° and 180°. These identified determinants influence the moment arm, GRF, or a combination of the two, therefore elevating KAMs; however, it appears that these biomechanical deficits are modifiable with appropriate training, feedback and conditioning [76–78]. Particularly, technical characteristics associated with safer side-stepping are as follows: reduced lateral foot-plant distances, thus lower hip abduction and orientating the foot closer to neutral with a mid-foot or forefoot placement strategy; minimising knee valgus and hip internal rotation angles and motion at IC and WA; avoiding and limiting lateral trunk flexion and attempt to maintain an upright trunk position or trunk lean into the intended direction; reducing the magnitude of GRF during WA in the plant foot, potentially by attenuation through increased knee flexion and emphasising a greater proportion of braking in the penultimate foot contact. The variables associated with increased KAMs support the commonly identified visual characteristics of ACL injuries including wide lateral foot plant with hip abduction, knee valgus and the trunk flexed and/or rotated towards the plant foot [2, 4–7, 79]; thus, strengthening the argument to avoid and limit these potentially hazardous alignments, motions and higher GRFs during rapid cuts. These determinants and biomechanical deficits can subsequently be used to identify athletes displaying “high-risk” patterns during screening and can also form the basis for ACL injury mitigation programmes. However, practitioners should be aware that some of the “high-risk” postures identified in this review are necessary for faster cutting performance (i.e. wide foot plant, decreased knee flexion); thus, practitioners should be conscious of the “performance injury conflict” when addressing certain high-risk postures, because athletes are unlikely to adopt “safer” movement strategies at the expense of faster performance. Appropriate strength and conditioning intervention to improve eccentric strength, and thus braking capacity, may offer the answer to this performance injury conflict, by concurrently reducing KJLs and performance times.

## Data Availability

Not applicable

## Abbreviations

3D: Three dimensional
ACL: Anterior cruciate ligament
COD: Change of direction
COM: Centre of mass
COP: Centre of pressure
GRF: Ground reaction force
HBF: Horizontal braking force
IC: Initial contact
KFM: Knee flexion moment
KAM/pKAM: Knee abduction moment/peak knee abduction moment
KEM: Knee extension moment
KIRM: Knee internal rotation moment
KJL: Knee joint load
PFC: Penultimate foot contact
WA: Weight acceptance
